# The role of budget impact in reimbursement decisions in The Netherlands: interviews with decision-makers and pharmaceutical industry representatives

**DOI:** 10.1007/s10198-025-01771-w

**Published:** 2025-04-09

**Authors:** Vivian Reckers-Droog, Joost Enzing, Werner Brouwer

**Affiliations:** 1 Erasmus School of Health Policy & Management (ESHPM), Erasmus University Rotterdam, P.O. Box 1738, 3000 DR Rotterdam, The Netherlands; 2 National Health Care Institute (ZIN), Diemen, The Netherlands

**Keywords:** Budget impact analysis, Cost-effectiveness analysis, Decision-making framework, Reimbursement decisions, A13: Relation of Economics to Social Values, D61: Allocative Efficiency · Cost–Benefit Analysis, D63: Equity, Justice, Inequality, and Other Normative Criteria and Measurement, I13: Health Insurance, Public and Private, I18: Government Policy · Regulation · Public Health

## Abstract

Economic evaluations of health technologies increasingly encompass a cost-effectiveness analysis (CEA) and a supplementary budget impact analysis (BIA) to inform reimbursement decisions on health technologies. Evidence from the Netherlands suggests that CEA requirements are consistent between the different stages of the decision-making process in the Netherlands, while BIA requirements are not. It remains unclear why aspects of BIAs vary in form and importance across decision stages, and why BIA results do not have a clear and consistent relationship with CEA results. Therefore, this study aimed to obtain further insight into the role of budget impact in the different stages of the decision-making process in the Netherlands, and into the experiences of decision-makers that may explain the variation in use of BIA across these stages. To meet this aim, we conducted semi-structured interviews with 12 decision-makers and 3 pharmaceutical industry representatives. Our findings indicate that BIAs serve multiple purposes depending on the responsibilities and needs of decision-makers in a specific decision stage. Each purpose may be relatively well-defined, and decision-makers seemingly have a clear understanding of the evidence on (aspects of) budget impact required for achieving their specific purpose. For example, the selection of pharmaceuticals for assessment is based on the maximum financial risk associated with reimbursement, discarding evidence on savings and substitution effects in other budgets and sectors, while these broader healthcare and societal elements are included during the appraisal stage. Hence, a clear framework for the consistent use of evidence on budget impact across decision stages has not yet been established.

**Supplementary Information:**

The online version contains supplementary material available at 10.1007/s10198-025-01771-w.

## Introduction

In many countries, economic evaluations of new health technologies are used to inform resource allocation decisions. These evaluations increasingly encompass a cost-effectiveness analysis (CEA; broadly including cost-utility analysis) to assess the value for money of publicly reimbursing a health technology, and a supplementary budget impact analysis (BIA) to assess the financial consequences of reimbursing and diffusing a technology within the healthcare system [1, 2]. In these countries, guidelines and requirements for performing a CEA, and well-defined decision rules based on the outcome of a CEA, are often in place. Typically, these stipulate that the incremental cost-effectiveness ratio (ICER) of a health technology should fall below some relevant monetary threshold for the technology to be eligible for reimbursement [3]. In addition, guidelines and requirements for performing a BIA have been developed [1, 2, 4]. However, well-defined decision rules based on the outcome of a BIA, as well as on developing a clear understanding of the role of a BIA and its relationship with a CEA in the decision-making process, arguably are largely lacking [5, 6].

**Fig. 1:**
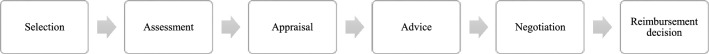
Stages of the decision-making process in the Netherlands [7, 8]


A recent review of policy reports and Ministerial correspondence was conducted to provide insight into the role of budget impact and its relationship with cost-effectiveness in the different stages of the decision-making process made in, what is known as, the closed entry system) in the Netherlands [7–9]. Besides the closed entry system, there is an open entry system in the Netherlands. Text Box 1 provides a brief description of the two entry systems and Fig. 1 presents a graphical representation of the decision stages.

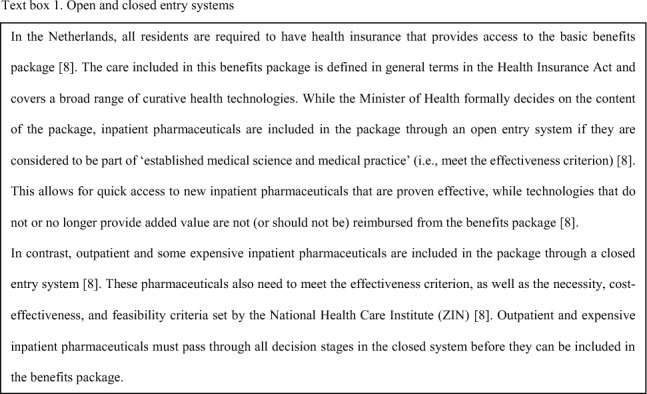


The results of the recent review indicated that the budget impact and cost-effectiveness of a health technology (mostly of pharmaceuticals, but increasingly also of other types of technologies [9]) are both considered relevant by decision-makers, in each stage of the decision-making process [7, 8]. Indeed, the budget impact and cost-effectiveness of a health technology are considered relevant from the stage in which a health technology is being selected for a full and systematic assessment of evidence on its necessity (operationalised as disease severity[10]), effectiveness, cost-effectiveness, and the feasibility (operationalised inter alia as budget impact) of its reimbursement, to the stage in which the Minister of Health ultimately makes a reimbursement decision [7, 8].

Furthermore, the results of the review indicated that the requirements for evidence of a health technology’s cost-effectiveness and the application of the related decision rule were quite consistent across the different decision stages [7]. However, this consistency did not hold for budget impact [7]. Specifically, the definition of budget impact and the related evidence requirements were found to vary across decision stages [7]. Furthermore, some important aspects of budget impact—such as substitution and saving effects—were found to be considered in the assessment and appraisal stages but not in the negotiation and final decision stages [7]. Some other aspects of budget impact—such as other medical indications for which the technology is already reimbursed—were found to be considered in these later stages but not (assessed and appraised) in earlier stages [7]. It remained unclear from the results of the review why BIAs are not aligned with CEAs (e.g., in terms of the underlying economic and clinical assumptions), why aspects of BIAs vary in form and importance across decision stages, and why the results of BIAs do not (yet) have a clear and consistent relationship with the results of CEAs in the decision-making process in the Netherlands [7].

Nonetheless, decision-making practice shows that the assessment and appraisal of the outcome of a BIA may depend (at least in part) on the outcomes of a CEA, and vice versa [7]. For example, members of the Insured Package Advisory Committee (ACP) openly debated in the appraisal stage of some new health technologies whether it was justifiable to reimburse a health technology with an unfavourable ICER on the grounds that it was indicated for a small patient group and, therefore, had a low budget impact [11, 12]. Conversely, the ACP debated whether it was justifiable to not reimburse a technology with a similarly high ICER on the grounds that it was indicated for a large patient group and, therefore, had a high budget impact [7]. This highlights that different characteristics of health technologies, including their budget impact, are (in some way) considered in reimbursement decisions in the Netherlands. However, the exact definition, operationalisation, and role of budget impact throughout the decision-making process remains unclear and seemingly varies from stage to stage, and from case to case. Obtaining better insight into such variation may be important for stakeholders, amongst whom manufacturers, patients, and members of the public.

The aim of the current study was to obtain further insight into the role of budget impact in the different stages of the decision-making process and the experiences of decision-makers that may explain the variation in use of BIA across these stages in the Netherlands. To meet this aim, we interviewed decision-makers involved in different decision stages who could narrate first-hand about the role and weighting of (aspects of) budget impact in reimbursement decisions in the Netherlands. Given that it is predominantly pharmaceuticals that currently undergo the different decision stages, we also interviewed pharmaceutical industry representatives (hereafter: representatives) to obtain insight into their perspective on the role of budget impact. The results of this study may be relevant for those seeking to clarify the role of BIA in decision making, increase the transparency and consistency of reimbursement decisions, and manage the expectations of stakeholders regarding such decisions in healthcare.

## Methods

### Sample and data collection

We conducted semi-structured online interviews with 12 decision-makers and 3 representatives in the Netherlands (n = 15). Two interviews were conducted with two participants jointly, resulting in a total of 13 interviews conducted between June and October 2022. The interviews were conducted in the Dutch language (nine by the first and second author, and four by the second author) and recorded using Microsoft Teams. The recordings were transcribed verbatim by subcontractors and assessed for their quality before the analysis.

The decision-makers were purposively sampled based on their specific expertise on budget impact and the weighting of evidence on (aspects of) budget impact in one of the decision stages in the Netherlands. We did not interview the incumbent Minister of Health who ultimately decides on reimbursement of a health technology in the final decision stage as his decision would primarily be informed based on the results and recommendations from the preceding stages. The representatives were purposively sampled based on their expertise with regard to the experiences and interest of manufacturers in relation to the decision-making process the Netherlands. As such, these participants had more general expertise on the role of budget impact in the different decision stages.

Before we conducted the interviews, we informed the participants in writing about the aim and procedure of this study, provided them with the interview protocol described below to facilitate their preparation, and obtained their written consent for recording the interviews and using their anonymised data (without compensation) for meeting the study aim. We agreed with all participants that we would not report on the details of specific reimbursement cases and with participants identified (ID) as ID3, ID4, ID10, and ID11 (see Table 1 in the Results section) that we would not directly quote them in the paper. Note that we do not present any sociodemographic characteristics of the participants in the Results section to safeguard their anonymity. We obtained ethical approval for conducting this study from the Research Ethics Review Committee of Erasmus School of Health Policy & Management (ETH2122-0624).

**Table 1:** Interview and participant characteristics

Interview	Decision stage	Participant ID
1	Assessment	1
2	Appraisal	2
3	Negotiation	3
	Negotiation	4
4	Selection	5
5	Assessment	6
6	Appraisal	7
7	NA	8
8	Negotiation	9
9	NA	10
	NA	11
10	Assessment	12
11	Assessment	13
12	Advice	14
13	Assessment	15

ID, identification number; NA, Not Applicable

### Interview protocol

The interviews were standardised by using the interview protocol presented as Supplementary Material S1. Each interview started with an introduction by the interviewer(s) to the general aim and procedure of the study, and a clarification of the use of the terms ‘budget impact’ and ‘decision-making process’ to avoid any miscommunication during the interview. The interview with decision-makers then proceeded with some general questions on the (years of) expertise regarding a specific decision stage and, subsequently, with specific questions on the role of budget impact in that stage and the transfer of evidence on (aspects of) budget impact from one decision stage to the next. The interviews with representatives were similarly structured; however, the questions on budget impact focused on the overall decision-making process, rather than on one decision stage.

### Data analysis

The first two authors individually coded text blocks in the transcripts using an inductive approach. The first author translated the text blocks from Dutch to English and integrated the blocks into narratives that described the role and weighting of (aspects of) budget impact in each stage of the decision-making process. All narratives were cross-checked and refined by the second author, and those of participants with ID3, ID4, ID10, and ID11 were paraphrased such that we preserved the narrative quality of the text without using direct quotations. We used square brackets to indicate where we have omitted or added words to increase the readability of the narratives.

## Results

Table 1 presents the interview and participant characteristics. The sample consisted of 15 participants (i.e., 12 decision-makers and 3 representatives), of whom 7 were female. The decision-makers on average had 6 years of expertise (range 3–12 years) in the respective decision stage and the representatives on average had 3 years of expertise (range 2–4 years) in their current role. Of the decision-makers, one was involved in the selection stage, five in the assessment stage, two in the appraisal stage, one in the advice stage, and three in the negotiation stage of the decision-making process. Nine currently worked at the National Health Care Institute (ZIN) and three at the Ministry of Health, Welfare and Sport (VWS).

Table 2 presents a summary of the responsibilities, role of budget impact, and main experiences of the decision-makers in each of the decision stages. In what follows below, we report on the results from the interviews with decision-makers in the order of the decision stages as presented in Fig. 1. We present the results from the interviews with representatives in text boxes and italics to clearly distinguish them from the results of the interviews with decision-makers and highlight their contrasting perspectives. The results focus on pharmaceuticals considering that they predominantly go through the whole decision-making process. When relevant, we explicitly refer to other types of health technologies and clarify narrative excerpts using policy reports or Ministerial correspondence on the role of budget impact in reimbursement decisions in the Netherlands.

**Table 2:** Summary of the responsibilities, role of budget impact, and main experiences of decision-makers in each decision stage

Decision stage	Responsibility of decision-makers	Role of budget impact	Main experiences of decision-makers
Selection	Members of the Horizonscan team at ZIN identify and select inpatient pharmaceuticals for a full and systematic assessment	Members of the Horizonscan team focus on the maximum financial risk (which they estimate in terms of expected gross expenditures) associated with the reimbursement of pharmaceuticals from the pharmaceutical budget	Decision-makers base their decision on evidence on expected gross expenditures and may largely discard evidence on potential savings and substitution effects
Assessment	Pharmacoeconomic advisors at ZIN assess the effectiveness, necessity, and cost-effectiveness of selected inpatient and outpatient pharmaceuticals, and the feasibility of their reimbursement	Budget impact is a key factor in assessing the feasibility of reimbursement	Decision-makers face challenges with complex BIAs and incentives for manufacturers to misestimate
Appraisal	Members of the ACP appraise the societal desirability of reimbursing pharmaceuticals based on justice and solidarity principles	Budget impact is considered relevant, especially in relation to the opportunity costs of reimbursing pharmaceuticals and the related risk of crowding out other health technologies. Savings and substitution are considered	Decision-makers consider the broader societal impact and fairness of the budget impact of reimbursing pharmaceuticals, also in relation to the number of indications for which pharmaceuticals are (or likely will be) reimbursed
Advice	The Board of Directors of ZIN advises the Minister of Health on reimbursement of pharmaceuticals based on their review of the assessment and appraisal reports	Budget impact is considered in relation to the effectiveness, necessity, and cost-effectiveness of pharmaceuticals, as well as in relation to the societal acceptability of their prices	Decision-makers weigh and balance multiple arguments and relevant uncertainties in their advice, also in relation to the price of pharmaceuticals and the societal desirability of their reimbursement
Negotiation	The Minister of Health decides on reimbursing pharmaceuticals and may initiate negotiations with manufacturers before making these decisions	Budget impact influences negotiations, incorporating considerations for volume discounts and savings in relation to the pharmaceutical budget, with the ultimate aim of meeting the cost-effectiveness criterion	Decision-makers navigate confidential negotiations and information asymmetry with manufacturers

ACP, Insured Package Advisory Committee; ZIN, National Health Care Institute

### Selection stage

In this stage, members of the Horizonscan team gain insight into the health technologies that can be expected to enter the Dutch market within two years. The Horizonscan team was initially established by the Ministry of VWS to identify and select pharmaceuticals that were candidates for the ‘lock’ [8], which was implemented to prevent expensive inpatient pharmaceuticals from entering the basic benefits package without any formal assessment on economic and clinical evidence. This task has been fully assigned to ZIN since January 2017.

The Horizonscan team identifies and selects pharmaceuticals that need to undergo a full and systematic assessment of evidence on the necessity, effectiveness, cost-effectiveness, and feasibility (including budget impact) of reimbursement. This selection is “influenced by capacity constraints” (ID9) and based on the “maximum financial risk that can be expected in case of reimbursement” (ID5). For inpatient pharmaceuticals the threshold (to be selected for assessment) for maximum financial risk is currently set at an expected gross expenditure on the pharmaceutical of more than €20 million per year for one or more medical indications *or* an expected gross expenditure of €10 million per year in combination with treatment costs per patient of more than €50,000 per year for a single indication [13]. For outpatient pharmaceuticals this threshold is set at an expected gross expenditure on the pharmaceutical of €10 million per year, *or* between €1 and €10 million per year in combination with treatment costs per patient of more than €50,000 per year [13].


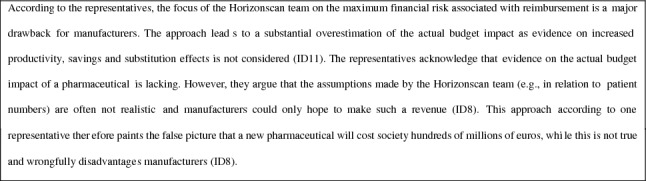


The Horizonscan team considers “what is already on the market, what the alternatives are, and which alternatives can be expected” (ID5). However, considering that the team focuses on the maximum financial risk “it is considered less important whether a pharmaceutical actually substitutes another pharmaceutical” (ID5). What is considered important is “whether there is any evidence on clinical value and, therefore, whether it can be expected that a pharmaceutical is going to be used at all in an inpatient or outpatient setting […] that is often not clear [in this stage]” (ID5).

There are currently no guidelines for determining what evidence is relevant in the selection of a pharmaceutical for assessment. Rather, this is determined based on “discussions with experts, medical specialists, hospital pharmacists, and representatives of patient organisations in [one of] the [eight disease-domain specific [14]] working groups” (ID5). “Manufacturers try to substantiate claims, but [the team] relies on experts for this” (ID5). Nonetheless, assumptions or expectations that the team cannot substantiate based on national or international sources are typically excluded [14]. For example, “experts may say that there are 100 patients with a specific condition and that it can be expected that a pharmaceutical will be used for 25 percent of those patients” (ID5). The team may then decide “to leave it at those 100 patients, because the expectations are just too uncertain and [that percentage] cannot be substantiated” (ID5).

The Horizonscan team biannually reports to the House of Representatives on the new pharmaceuticals that can be expected to enter the market [8]. These reports are not only used by the Minister of Health for making decisions regarding the placement of pharmaceuticals in the ‘lock’, but also by “hospitals and health insurers for negotiating on the provision and compensation for provided care” (in, what is referred to as, the ‘open system’ in the Netherlands [8, 9]) (ID5). They are furthermore used by ZIN “for anticipating on the number of assessments that can be expected” (ID5). The selection of pharmaceuticals for placement in the lock is “really based on the financial risk of reimbursing a pharmaceutical, not on its cost-effectiveness or any characteristics of the disease […] that is something for later stages in the [decision-making] process” (ID5). The cut-off values used for determining the financial risk “may be arbitrary”, but “they are just a tool to identify the pharmaceuticals that are extremely expensive and need to be looked at with close attention […], often this concerns orphan drugs” (ID5). Some manufacturers “really try to stay below the cut-off values [and] health insurers would like to see that these ‘lock divers’ are selected [for assessment] regardless” (ID5). Ideally, “ZIN would, of course, also assess these pharmaceuticals” and “health insurers could explicitly ask ZIN to do this, but in practice that is possible for only one pharmaceutical per year, which is not much” (ID5).
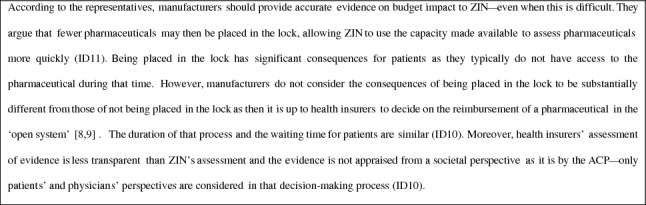


The Horizonscan team not only provides information to decision-makers in subsequent decision stages but also receives information (e.g., from Pharmacoeconomic advisors at ZIN involved in the assessment stage) based on which they update their reports. The team “makes use of the available information [and] if information on patient numbers or comparable pharmaceuticals is available, we aim to make use of those numbers because, ultimately, we try to make sure that we are not just putting down maximum numbers but that we are as objective as possible” (ID5).

### Assessment stage

In this stage, Pharmacoeconomic advisors at ZIN assess the scientific rigor of and assumptions underlying the evidence on evidence on the necessity, effectiveness, cost-effectiveness, and feasibility of reimbursement (including the budget impact), for which they are assisted by members of the independent Scientific Advisory Board (WAR) [9].

The advisors “first look at whether a pharmaceutical has any added therapeutic value in comparison to pharmaceuticals that are already reimbursed” (ID1). In case “a pharmaceutical is interchangeable with another pharmaceutical that is already reimbursed, no additional costs should be involved with reimbursement […] this has to be budget neutral” (ID1). If a pharmaceutical is not interchangeable, the advisors “check if the budget impact is more than 10 million euros as part of the feasibility criterion” (ID13). In case the “budget impact is below that cut-off value, it is less worthy of attention” (ID1). In some cases, “[the pharmaceutical] is above the cut-off value but cost-effective, in which case you also do not want to go through all the trouble of a full assessment” (ID1). However, in some other cases, “the budget impact may be below the cut-off value, but you still want to do a full assessment, for example, when a pharmaceutical is very expensive and only used by one or two patients per year” (ID1).

“Cost-effectiveness and budget impact, of course, go hand in hand” (ID1). For example, if ZIN “advises the Minister [of Health] to negotiate a lower price to bring down the ICER, you automatically also bring down the budget impact” (ID1). It would be possible to “more closely align [BIAs and CEAs] and see certain cost items reflected in a BIA if they are part of a CEA model but, ultimately, the BIA then almost becomes some kind of CEA, and it becomes difficult to determine the boundary between the two […] where does one type of analysis stop and the other begin?” (ID12). This is why “we agreed to just focus on the pharmaceutical budget” (ID12).

In contrast to the selection stage, “factors such as market share and speed of market penetration are [considered] important [in the assessment stage]” (ID1), also in relation to the “prescription behaviour of physicians and willingness of patients to switch to a different pharmaceutical” (ID12). “Strictly speaking, a BIA focuses on the pharmaceutical budget; however, if there are noteworthy costs that are related but fall outside that budget, they are included in a report” (ID 13). A BIA is “often very complex and advisors have internal rules on how to handle things, for example, in relation to the moment patients fall ill, their treatment adherence, and the costs associated with waste of resources” (ID12). Manufacturers “have incentives to underestimate the budget impact to be exempted from a CEA, but also to strategically overestimate it in view of price negotiations with [the Ministry of] VWS” (ID12). These incentives “are not always clear, and it can take a lot of effort to figure out how something really is, and [advisors] sometimes have to make quite some assumptions” (ID12).
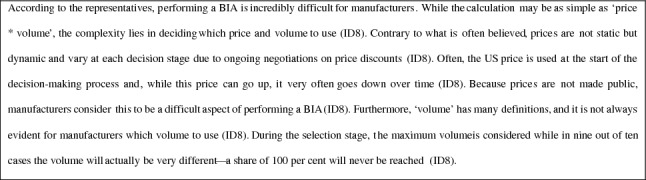


Preferably, the advisors would perform a full assessment of all health technologies that become available. They consider budget impact to be “highly important as it determines whether ZIN is going to perform an assessment at all, or whether this is left to health insurers [in the open system]” (ID15). It indeed “makes sense that budget impact would determine the agenda, but the cut-off value is too narrow and strict [and, consequently], we do not assess all technologies that we would want to assess and, sometimes, we assess technologies that we should not care about too much, which is suboptimal” (ID15). There are “other factors that are important, such as the treatment landscape but these are not stringent criteria” (ID15). “Thinking about it, stringent criteria are also not easy to deal with […] ideally, the entire healthcare system would be closed, and we would decide based on relevant, but more flexible, criteria which [new technologies] would be reimbursed” (ID15). “A little more flexibility would be nice; however, at the same time it is important to be transparent, consistent, and predictable for manufacturers, so they know where they stand” (ID15).

In the assessment stage, the advisors and WAR “assess the quality of the evidence on the therapeutic value and cost-effectiveness [of a technology] and in the next stage, the ACP assesses whether the price of a technology weighs up to its effectiveness” (ID15). In that context, “it matters whether a patient population is small or large, and it can be justified that you are stricter regarding what you are willing to pay when the budget impact is very high—regardless of the cost-effectiveness” (ID15). In case it is “incredibly high, it is reasonable to ask for a lower price […] if you consider the profit made by manufacturers, we should not pay the maximum of the cost-effectiveness threshold, and we can be explicit about using budget impact as an argument to limit our willingness to pay” (ID15). “This argument and [basically] all arguments other than cost-effectiveness—such as the crowding out of health care—have gained importance over the past couple of years due to the pressure on healthcare affordability and expenditures on expensive pharmaceuticals" (ID15).
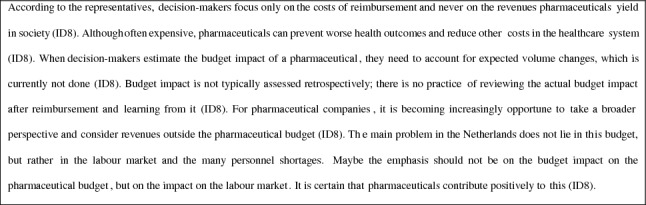


After the advisors have determined whether the obtained evidence can be considered “scientifically sound”, “the reports [of the advisors and WAR] go to the next [decision] stage” (ID1). Although “advisors and [members of] the WAR may have more thoughts on a dossier, [these thoughts] are not included in a report […] even when they are considered relevant [for the decision]" (ID6). That may be “surprising because members of the WAR are more than technical advisors—they might just as well have been members of the ACP” (ID6).

### Appraisal stage

In this stage, the ACP appraises whether reimbursement can be considered socially desirable based on the principles of justice and solidarity [9]. Often, “[evidence on] budget impact is the main reason for a reimbursement dossier to be appraised by the ACP, along with [evidence on] cost-effectiveness—be it unfavourable or otherwise” (ID7). The ACP “considers issues like ‘this is the fourth indication for a pharmaceutical and the manufacturer should have had the return on investment by now’” (ID1). The ACP’s “framework of arguments is broad, which can make the appraisal difficult, but their discussion is coherent, they look at the whole picture […] and the nice thing is that patients, manufacturers, and everyone with an interest can contribute to [their discussion], it is transparent for the outside world” (ID1). In their discussions, “budget impact is definitely a relevant factor in relation to the crowding out [of healthcare]; if you spend money on an expensive pharmaceutical, you can no longer spend it on extra childcare in a hospital, for example” (ID1). The exact role of budget impact “may differ between different reimbursement cases, but the process is the same for all pharmaceuticals; how it is deployed in the process is consistent” (ID1).

In the past, “the concept of budget impact was very unclear and the costs [of reimbursing a pharmaceutical], whether high or low, were considered irrelevant—as was the distinction between small and large patient groups” (ID7). The main concern was “whether the money was being spent efficiently” and, as such, the “number of patients and budget impact were not considered important at all and if it was, it was unclear how to estimate it […], there was no theoretical basis for it at all” (ID7). Nowadays, budget impact is considered important “because of the risk of making a wrong [reimbursement] decision” (ID7). From that perspective, “it can be understood why politicians do find budget impact important: when it is low, the consequences of making a wrong decision can more easily be compensated elsewhere” (ID7).

“Under the heading of solidarity”, budget impact will “of course be part of the ACP’s Framework of Arguments [Expensive Pharmaceuticals] that is currently being drafted” (ID7) [and was recently published] [12]. The ACP “has clear ideas about [budget impact]” and “is kind of sad about the cut-off values for the ‘lock’ because some pharmaceuticals do not come into the ACP’s focus even though they do incur significantly high costs” (ID7). Pharmaceuticals with a low budget impact are “relatively easily reimbursed, even when they are hardly effective” and the ACP “considers it unfair that [the budget impact of] a pharmaceutical is more likely to stay below the cut-off value when there are only two patients than when a condition is much more common” (ID7). Members of the ACP “do not necessarily consider it fair that people with a rare condition are favoured, they should not be disadvantaged, nor should they be advantaged [as compared to people with a common condition]” (ID7). A “strong incentive has arisen to focus on small diseases and marginal innovations that do not actually have to be assessed because of their limited budget impact, so we will just muddle through” (ID7). Indeed, “a crazy situation has developed, just look at the budget that we spend per patient—we spend a lot of money on [pharmaceuticals for] diseases that are rare. It is disconcerting to think that the pharmaceutical industry relies on addressing these diseases for its existence. This contradicts the fundamental principles of epidemiology, especially in a system with public [health] insurance, where the primary goal is to combat diseases that affect large numbers of people. What we aspire to achieve is to provide effective treatments to as many people as possible. However, we find ourselves directing resources towards a small group of patients for pharmaceuticals with limited treatment efficacy. It appears that we have inadvertently incentivised the pharmaceutical industry to shift its focus from addressing significant health issues to niche markets that involve treating rare diseases and charging exorbitant prices. This shift is primarily driven by political sensitivity to the budget impact of pharmaceutical” (ID7).
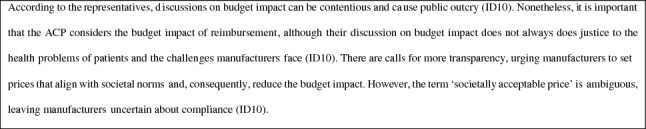


The ACP “considers the consequences of reimbursement for society, regardless of whether this concerns the pharmaceutical budget or any other budget” (ID7). When the budget impact is high, the ACP thinks about “what else can be done with that large sum of money” (ID7). The appraisal by the ACP “follows a set pattern, starting with the added [therapeutic] value of the pharmaceutical, whether the cost-effectiveness is unfavourable or favourable, whether the evidence is well founded, whether there are any uncertainties” (ID7). If the cost-effectiveness is unfavourable, there may be “reasons to accept this, for example, because there is no treatment available for patients yet or because their disease is very severe” (ID7). The appraisal of the ACP “almost always comes down to the advice to negotiate about the price [of the pharmaceutical]. This [advice] is not so much based on the budget impact [of the pharmaceutical], but on its cost-effectiveness […] there must be a price that makes the pharmaceutical cost-effective—that is the starting point” (ID7). Nonetheless, there recently was “a case where the pharmaceutical was cost-effective, yet the Minister [of Health] still initiated price negotiations on grounds of its high budget impact” (ID7).

The ACP considers it important that “a manufacturer—not society—should bear the risk of a potentially wrong decision, for example, in case there are many uncertainties about the pharmaceutical, for example, in terms of the effectiveness and patient numbers” (ID7). This “should be reflected in a lower price, and hence also in a lower budget impact” (ID7). As such, it could be that “the ACP appraises the budget impact of a pharmaceutical”, also because “you grant a manufacturer the possibility to recoup their investment given the number of patients that may use the pharmaceutical but, of course, a pharmaceutical company since long does not operate nationally, it operates internationally” (ID7). “If the number of patients is higher, the price of course should be lower in order to give it an honest reward […] so yes, these are all factors that are related to the budget impact of a pharmaceutical” (ID7). It is becoming increasingly important for the ACP to not only “consider the value of the pharmaceutical for patients, but also the reasonableness of the price that is being charged by the manufacturer, also in relation to the number of [previous] indications of the pharmaceutical” (ID7).
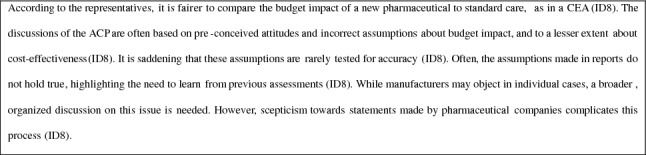


The ACP has “experimented with using multi-criteria decision analysis for weighting arguments in their appraisals but the conclusion was that it was not possible to always weigh budget impact with a specific factor” (ID7). There is “a relatively fixed protocol for the order in which arguments enter the ACP’s discussion, also in relation to previous reimbursement cases that share similarities with the current case” (ID7). It is “of course important that the ACP is reliable and consistent, but reimbursement cases are never identical” (ID7). Furthermore, the ACP “pursues a degree of heterogeneity in their appraisals […] manufacturers may consider this to be subjective, but there is a discernible pattern to the appraisals” (ID7). It is “important that all arguments can be related back to the four decision criteria [necessity, effectiveness, cost-effectiveness, and feasibility]” (ID7). In itself, “budget impact is not a criterion […], it is part of the feasibility criterion, but it can be used to be more critical of the other criteria” (ID7). In a meeting on the (recently published) Framework of Arguments Expensive Pharmaceuticals [12], the ACP was “struggling with the question of how exactly budget impact should fit into [their] considerations and then [they] literally said it like’ if [the budget impact] is high then that is a kind of red flag to look carefully at other arguments’. Of course, that has everything to do with the risk of making the wrong reimbursement decision, which is very high if the budget impact is high” (ID7). The ‘Framework of Arguments Expensive Pharmaceuticals’ was drafted, because the ACP has had discussions on expensive pharmaceuticals for some years now and want to move [their discussions] on to other issues that are important” (ID7). For example, issues relating to the “limited added [therapeutic] value of new pharmaceuticals […] as hardly any curing is currently taking place” and “for the same amount of money, we [as a society] can decide to keep a hospital open or prevent nurses from losing their jobs” (ID7).

### Advisory stage

In this stage, the Board of Directors of ZIN reviews the assessment and appraisal reports from the preceding two stages, based on which it advises the Minister of Health on the reimbursement of a health technology. Prior this review, the Board participates in private and public meetings of the ACP. During private meetings, the Board “takes part as observer and generally refrains from taking part in any discussion […] it is the ACP’s meeting, so the initiative always lies with them” (ID14). During public meetings, the Board “takes part as attendee” and outside meetings, the Board “can make inquiries with the ACP through Advisors [at ZIN] but only becomes actively involved when the reports are placed on their desks” (ID14).

The Board “increasingly has to deal with the uncertainty of cost-effectiveness estimates and the weighting of multiple arguments” (ID14). The Board “has been talking about this a lot and it is becoming increasingly difficult to make any trade-offs without a clear framework” (ID14). Currently, these trade-offs “depend too much on how the estimates are interpreted and on what value is attached to them, because of which too many things get mixed up” (ID14). “There are, so to speak, so many factors, so many considerations that weigh in that it is difficult to reduce it to a clear policy of ‘this is how we do it in the Netherlands’[…] that process just is not black and white anymore” (ID14). The Board “wants to know which factors are considered by the ACP when [its members] talk about the costs and effectiveness [of a technology], and what the uncertainties are in that respect” (ID14). “The public has the right to put the consequences of those uncertainties back on the manufacturer” (ID14). In some cases, the Board is “of course willing to take more risks, but the process of weighting relevant arguments should be the same between cases—even when the weight of those arguments is different” (ID14). This is “a matter of principle […] you could say that [the Board] should be stricter when the budget impact is high, but the weighting process should be the same—regardless of the size of the budget impact” (ID14).

The Board “believes it is good that the ACP makes a statement on the costs of reimbursement” (ID14). However, “it seems to have become mostly an economic discussion, while the public should understand that [the Board] says ‘well, this is cost-effectiveness, this is budget impact, and this is the necessity’ and ‘these are the reasons why this trade-off is made’” (ID14). It “is often communicated to the public that ‘it is too expensive, and that [pharmaceuticals] should be made cheaper’, which may give a wrong impression” (ID14). The Board should “explain more clearly that that ‘these are the costs, these are the effects, and those two are out of balance’ so that when the Board says that ‘the price is too high’, the public knows that it has also taken a technology’s effectiveness into consideration” (ID14). The Board is “increasingly confronted with the power of the media, patients, and manufacturers and what you see is that the public is paying more and more for less [health] effects, all while uncertainties are increasing” (ID14).
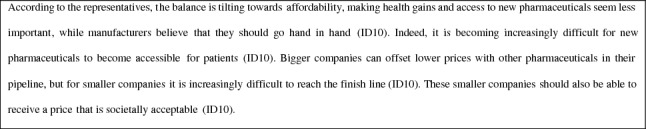


“[The focus on] appropriate care [i.e., care that adheres to four specific principles [15]] includes the question of whether we should want [to reimburse] all this […] if you look at those expensive pharmaceuticals, at what they cost, maybe we should not want it all, which means we must take a different position with respect to pharmaceuticals and manufacturers” (ID14). “It is fair that manufacturers have a business model where they invest and get something in return but the revenue model behind it is exceeding all normal values and is not sustainable” (ID14).” “The important question that we must address in the coming time is ‘can we and should we want to bear those costs?’” (ID14). “The role of the Board is to think about this, about where all this is leading to, and to address this with the ACP and the WAR” (ID14).
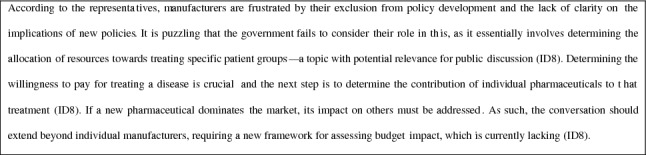


The Board includes “all trade-offs and relevant considerations in its reimbursement advice to the Minister of Health [and] from then on it is only about finances and negotiations” (ID14). “The question is whether you should leave that part of the process to the government or whether an independent authority should manage the basic benefits package’” (ID14). The Board is “increasingly open to taking on that responsibility, as it allows for keeping reimbursement decisions out of the political arena, which makes the process much easier” (ID14). “You need a political decision on the applied methodology, not on the reimbursement of single pharmaceuticals” (ID14). What the Board encounters in decision-making practice, is that “political lobbying is going on, especially when patients ‘have a face’ […] you then know beforehand that there will be a political debate about the decision” (ID14). “As a society, we fell into the trap of moving from cost-based to value-based decision making and we never dare to say ‘no’ to a manufacturer and then, no matter what framework you put in place, you will always come up short [and] manufacturers will always be best off, in whatever negotiation” (ID14). “We would have less of those discussions if we had kept ourselves to our own limits […] we opened the floodgates by always saying ‘yes’” (ID14).

### Negotiation and decision stages

In this stage, the Minister of Health decides either to directly reimburse a pharmaceutical—or other type of health technology, although this does not (yet) happen very often (ID9)—or to initiate negotiations on a financial arrangement with the manufacturer based on the advice of the Board of Directors of ZIN. The Minister of Health annually reports on new and ongoing financial arrangements with manufacturers (also in terms of savings on expenditures) without disclosing any confidential information to the House of Representatives [16].
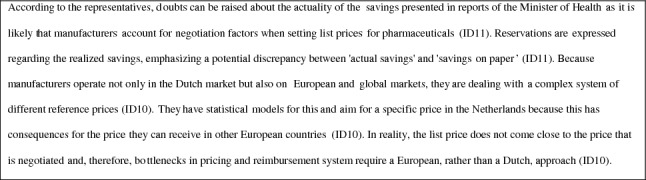


When negotiations are initiated, the Bureau Financial Arrangements Pharmaceuticals (BFAG) of the Ministry of VWS adheres closely to ZIN’s advice (ID4). However, it should be noted that ZIN’s advice always concerns one pharmaceutical and BFAG may also take a second or third pharmaceutical into account that is available for the same medical indication (ID4). The ACP may suggest that the BFAG negotiate an additional price discount if other pharmaceuticals are anticipated to enter the market, consistent with market forces (ID4). However, the BFAG does not aim to position itself as market regulator (ID5). The BFAG acknowledges that manufacturers are entitled to set high prices for pharmaceuticals, and it neither wants to control them nor has the authority to regulate them (ID5). Therefore, the BFAG focuses on the expenditures on the pharmaceuticals instead (ID5).

Price negotiations may also occur in relation to reimbursing a pharmaceutical for a less severely ill subgroup of patients, after price negotiations for a very severely ill subgroup of patients are concluded (ID5). In such cases, it can be argued that the price should be reduced to the production cost of the pharmaceutical, although manufacturers do not disclose those exact costs (ID3). In addition to cost-effectiveness, budget impact—or rather, the expected gross expenditures on a pharmaceutical—may also be relevant in negotiations. For example, when a pharmaceutical is expected to be used in treatment of large numbers of patients, negotiations on volume discounts may become relatively more important than those on a pharmaceutical’s price (ID4).
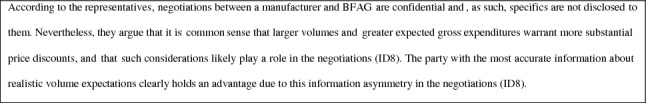


Cost-effectiveness may be the most important criterion on which negotiations are based as it defines the maximum price used by ZIN in their advice, sometimes leading to negotiations on a price reduction of 80 per cent (ID5). Whether this is realistic and achieved is classified (ID4). It should be noted that a cost-effective price is not necessarily considered acceptable in negotiations as there may still be a large producer surplus, and society should not pay too much for a pharmaceutical if that is not necessary (ID9). Manufacturers frequently use arguments in relation to substitution and saving effects in negotiations (ID3). They may argue that reimbursement of their product will (also) result in substitutions and savings in other parts of the healthcare system (ID4). However, BFAG focuses only on the budget for pharmaceuticals and broader savings—which are highly uncertain (ID3)—are already included in the cost-effectiveness of their product (ID4). Manufacturers seemingly assume that any potential savings will accrue to them (ID4). There may be indeterminate standards regarding the profit manufacturers should be able to make—even though pharmaceutical companies are commercial entities and, as already mentioned—BFAG is not a market regulator (ID4). Indeed, BFAG is responsible for optimizing the allocation of healthcare resources for society and, even in case a pharmaceutical is cost-effective, its price may not be considered acceptable from a societal perspective (ID3). Currently, there is no scientifically substantiated definition of what constitutes a reasonable or excessive price, nor is there a clear framework for determining what price is reasonable in relation to sales or expenditures on a specific pharmaceutical (ID9). There are no policies mandating manufacturers to offer greater transparency beyond a certain price level. While greater transparency would, at least in theory, strengthen the negotiation position of BFAG, it would also enhance predictability in negotiations for manufacturers (ID9). In principle, BFAG employs the same line of argumentation in each negotiation case, irrespective of whether a pharmaceutical is indicated for a small or large group of patients, or for patients with a common or rare disease (ID3). However, their negotiation strategy is dependent on the specific characteristics of the pharmaceutical, patients, and disease and, therefore, may differ between cases (ID3). For example, in some cases, the volumes are smaller, there is little or no competition from other manufacturers, and there is more political pressure and lobbying for pharmaceutical reimbursement (ID3). In those cases, negotiations on financial arrangements can be difficult (ID3).
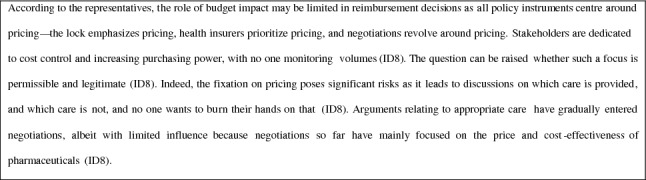


The decision-making framework is currently well-defined for cost-effectiveness; however, the framework remains unclear for (the different aspects of) budget impact (ID4). Further developing the framework and explicating the trade-offs between different decision criteria and between different aspects of budget impact will be useful, especially when based on quantitative evidence on social values in this context (ID4).

## Discussion

The aim of this study was to obtain further insight into the role of budget impact in the different stages of the decision-making process and the experiences of decision-makers that may explain the variation in use of BIA across these stages in the Netherlands. Our main findings indicate that BIAs seemingly serve multiple purposes depending on the responsibilities and needs of decision-makers in a specific stage of the decision-making process. Our findings furthermore indicate that each of these purposes may be relatively well-defined, and that decision-makers have a relatively clear understanding of the evidence on (aspects of) budget impact required for achieving their specific purpose. For example, the purpose of decision-makers in the selection stage is to identify and select pharmaceuticals for a full and systematic assessment of evidence based on the maximum financial risk that can be expected in case of their reimbursement. Hence, they require evidence on expected gross expenditures and may largely discard evidence on any potential, but uncertain, savings and substitution effects. Yet they ostensibly face challenges in determining how information on budget impact relates to the final reimbursement decision and how (aspects of) budget impact should be weighted in relation to the other decision criteria. No clear framework has yet been established that allows for a consistent use of information on budget impact across the different decision stages.

Nonetheless, it may reasonably be expected that achieving the purpose of a specific decision stage contributes to achieving the purpose of a subsequent stage—ultimately all serving the purpose of informing the reimbursement decision in the final stage. This is seemingly the case in the assessment, appraisal, and advisory stages where evidence on the budget impact of reimbursement is assessed and appraised, and subsequently incorporated in the reimbursement advice to the Minister of Health. However, our findings indicate that the BFAG may focus more on the evidence provided on the cost-effectiveness of a health technology and the recommendations of the ACP than on some (potentially important) aspects of its budget impact in their negotiations with manufacturers. For example, the BFAG may largely discard evidence on savings and substitution effects, as these may fall outside the pharmaceutical budget. Furthermore, the BFAG may consider other medical indications for which the technology is already reimbursed in negotiations, whereas the evidence is assessed and appraised for a single indication rather than multiple indications in preceding decision stages. Factors such as patient numbers and other medical indications are currently no formal decision criteria (in earlier stages), but our findings indicate that such factors may still influence the BFAG’s negotiations with manufacturers, even when a health technology is cost-effective. This also provides more perspective on previous findings suggesting that the interpretation of the outcomes of CEAs is relatively consistent between the different stages of the decision-making process [7]. Indeed, our findings indicate that not only the assessment and appraisal of the outcome of a BIA may depend on the outcome of a CEA, but that the reverse is also true. This implies that not only the role of BIA but also the role of CEA may not be fully clear and uniform in the (different phases of the) decision-making process in the Netherlands. Further research is warranted to obtain additional insight into the role of each individual criterion in final decisions, as well as the trade-offs between the different decision criteria and the preferences of relevant stakeholders in this context.

We would like to note that the BFAG may have good reasons for maintaining some confidentiality in the variety and relative weight of the factors they consider relevant in their negotiations with manufacturers. Nevertheless, by establishing a coherent framework for their negotiations, the BFAG may not only secure their strategic interests but also provide a roadmap for consistent and effective negotiations. Indeed, when the BFAG increases their transparency regarding the specific evidence they require and the importance attached to various factors, manufacturers may be more likely to see the value in providing relevant data.

## Conclusions

In conclusion, our findings indicate that BIAs serve multiple purposes depending on the responsibilities and needs of decision-makers in a specific stage of the decision-making process. Each of these purposes may be relatively well-defined and decision-makers seemingly have a clear understanding of the evidence on (aspects of) budget impact required for achieving their specific purpose. Nonetheless, a clear overall framework has not yet been established that allows for a consistent use of evidence on budget impact across the different decision stages. Decision-makers may discard evidence on savings and substitution effects and consider other medical indications in later stages, even when a health technology is cost-effective. This implies that the role of BIAs is not yet fully established in the Netherlands and there is value in exploring this further.

## Supplementary Information

Below is the link to the electronic supplementary material.

Supplementary file1 (DOCX 58 KB)

## Data Availability

The data are available from the authors upon reasonable request.
