# Supplementary material for: The role of budget impact in reimbursement decisions in The Netherlands: interviews with decision-makers and pharmaceutical industry representatives
Source: Eur J Health Econ. 2025 Apr 9;26(8):1333–45. doi: 10.1007/s10198-025-01771-w (PMC12572102; doi:10.1007/s10198-025-01771-w)
Supplement: Supplementary file 1 — Supplementary file1 (DOCX 58 KB) [file 10198_2025_1771_MOESM1_ESM.docx]

# **Supplementary Material S1**

**Interview protocol**

1. Welcome participant
2. Check informed consent form and approval for recording the interview
3. Start recording
4. Explain and check understanding of aim and procedure of study
5. Explain procedure of semi-structured interview and use of prompts (e.g., ‘Can you elaborate on this?’ and ‘Can you think of an example?’)
6. Clarify definitions used during interview:

- Budget impact: When we talk about budget impact, we mean the expected change in healthcare expenditure relating to the reimbursement and diffusion of a health technology within the healthcare system.
- Decision-making process: When we talk about the decision-making process, we mean the full process of selecting a health technology for a full and systematic analysis of evidence on its necessity, effectiveness, and cost-effectiveness, and the feasibility of its reimbursement, the assessment and appraisal of the evidence, the reimbursement advice of the National Health Care Institute (ZIN) to the Ministry of Health, Welfare and Sport (VWS), negotiations on a financial arrangement between the Ministry of VWS and manufacturers and, finally, the reimbursement decision made by the Minister of Health. This process consists of the six stages shown in the Figure below.

6. Check whether the definitions are clear and answer any questions before proceeding with the questions below.

| **Decision-makers** | **Pharmaceutical industry representatives^a^** |
| --- | --- |
| **General questions** | |
| 1. How would you describe your involvement in the reimbursement decision-making process in the Netherlands? | √ |
| 2. In which stage of the decision-making process are you involved? | √ |
| 3. How many years have you been involved in that stage? | √ |
|  |  |
| **Questions on budget impact** | |
| 4. What aspects of budget impact are considered relevant in the decision stage in which you are involved? | √ |
| 5. How is it decided (e.g., based on which policy rule or criteria) that those aspects are relevant? | √ |
| 6. Are other aspects of budget impact also considered? Please explain why (not)? | √ |
| 7. How is evidence on (aspects of) budget impact used (e.g., collected, assessed, or appraised) in the decision stage in which you are involved? | √ |
| 8. To what end is evidence on (aspects of) budget impact used in the decision stage in which you are involved? |  |
| 9. To what extent are market conditions (e.g., in relation to saving and substitution effects) considered in the stage in which you are involved? | √ |
| 10. How is evidence on (aspects of) budget impact transferred from the previous stage to the stage in which you are involved (if applicable)? |  |
| 11. How is evidence on (aspects of) budget impact transferred from the stage in which you are involved to the next stage (if applicable)? |  |
| 12. How—and to what end—will decision-makers in the next decision stage use the evidence that is transferred from the stage in which you are involved? |  |
| 13. To what extent is the transferred evidence on (aspects of) budget impact sufficient for meeting the aim of the decision stage in which you are involved? Can you think of any requests for additional evidence? If so, what type of evidence did this concern and how, and by whom was this evidence provided? |  |
| 14. How is evidence on cost-effectiveness (including evidence on the disease severity of patients) used in the decision stage in which you are involved? How does this relate to the use of evidence on (aspects of) budget impact? | √ |

^a^ Focus questions for pharmaceutical industry representatives on a specific decision stage when relevant, otherwise focus on the decision-making process in its entirety.

**Interview protocol**—**continued**

1. Is there anything we have not yet discussed that could still be relevant for our study?

2. Thank participant for their cooperation

3. Stop recording
